# STAFFING AND CARE QUALITY OF NURSING HOMES IN KOREA

**DOI:** 10.1093/geroni/igz038.2583

**Published:** 2019-11-08

**Authors:** Hee Seung Lee

**Affiliations:** National Health Insurance Service, Health Insurance Policy Research Institute, Wonju, Korea, Republic of

## Abstract

Staffing has been regarded as the most important factor for the quality of care service in nursing homes. Korea introduced Long-Term Care Insurance (LTCI) in 2008. The payment system of LTCI has incentivized LTC facilities based on the staffing level of LTC facilities. This study aims to investigate whether staffing is associated with quality of care. The effect of staffing on care quality was assessed using ordered logit analysis. Staffing data in 2015 were retrieved from claim data in the National Health Insurance Service. The publicly reported care service quality grade in 2015 was used as a proxy for care quality. Staffing of registered nurses (RN) and social workers were strongly associated with the care quality. As the number of RNs per residents additionally increased, the LTC facilities were more likely to receive better grades (OR=16851.54, p<0.000). The effect of social workers’ staffing was significant for the care service quality, even though the effect size of smaller than that of RNs (OR=345.87, p<0.000). However, staffing in other professions such as nurse assistants (NA) and personal care workers (PCW) was insignificantly associated with care quality. The effect of staffing on service quality might not be profession-neutral. RN staffing affects most in care quality in Korea. Still, the possibility remains that PCWs or NAs could serve for indirect care services such as cooking or cleaning because of short staffing in indirect care services. This finding could be considered when designing financial incentives for nursing homes in Korea as well as other countries.

